# Discovery of Intramolecular Signal Transduction Network Based on a New Protein Dynamics Model of Energy Dissipation

**DOI:** 10.1371/journal.pone.0031529

**Published:** 2012-02-20

**Authors:** Cheng-Wei Ma, Zhi-Long Xiu, An-Ping Zeng

**Affiliations:** 1 Institute of Bioprocess and Biosystems Engineering, Hamburg University of Technology, Hamburg, Germany; 2 School of Life Science and Biotechnology, Dalian University of Technology, Dalian, China; Semmelweis University, Hungary

## Abstract

A novel approach to reveal intramolecular signal transduction network is proposed in this work. To this end, a new algorithm of network construction is developed, which is based on a new protein dynamics model of energy dissipation. A key feature of this approach is that direction information is specified after inferring protein residue-residue interaction network involved in the process of signal transduction. This enables fundamental analysis of the regulation hierarchy and identification of regulation hubs of the signaling network. A well-studied allosteric enzyme, *E. coli* aspartokinase III, is used as a model system to demonstrate the new method. Comparison with experimental results shows that the new approach is able to predict all the sites that have been experimentally proved to desensitize allosteric regulation of the enzyme. In addition, the signal transduction network shows a clear preference for specific structural regions, secondary structural types and residue conservation. Occurrence of super-hubs in the network indicates that allosteric regulation tends to gather residues with high connection ability to collectively facilitate the signaling process. Furthermore, a new parameter of propagation coefficient is defined to determine the propagation capability of residues within a signal transduction network. In conclusion, the new approach is useful for fundamental understanding of the process of intramolecular signal transduction and thus has significant impact on rational design of novel allosteric proteins.

## Introduction

The structure of a protein is the basis for understanding its function. However, the function is ultimately governed by its dynamics in most cases. Proteins are inherently dynamical molecules that undergo structural fluctuations over a wide range of timescales [1], [2]. Therefore, a thorough knowledge of the principle(s) governing protein dynamics is of fundamental importance for functional study and design of new protein functions. As a classic model for understanding the relationship(s) among protein structure, dynamics and function, allosteric proteins have attracted large attention for decades (for recent reviews see [3], [4], [5], [6]). The concept of protein allostery began with the Monod-Wyman-Changeux (MWC) model (also known as the concerted model or symmetry model) [7] and the Koshl-Némethy-Filmer (KNF) model (also known as the sequential or “induced fit” model) [8], which sought to account for allostery based on gross properties of the transition between two well-defined end-states. More recent thermodynamic models of allostery emphasize population shifts in conformational ensembles [9], [10], [11]. There has been experimental evidence that alternate allosteric states are simultaneously populated in solution [12], [13]. Furthermore, intramolecular signal transduction has been proposed as a key concept of protein allostery [4], [5], [14] and successfully used for redesign of protein functions [15], [16]. Nevertheless, none of these models describe how the signal is transferred from the regulatory site to the active site upon binding of an effector to the allosteric site. Recently, we proposed a new protein dynamics model [17], which considers the signalling process as the result of energy dissipation.

Perturbation dynamics has been used to discover discrete breathers in protein structures [18], [19] and long-range energy transfer in proteins [20]. In these studies, it was shown that high amounts of energy may pop up in (or near) enzyme active sites, as a consequence of large and long-lived thermal fluctuations of nonlinear origin. Moreover, perturbation waves can also be applied in proteins and protein networks in signaling and drug design [21], [22]. By simulating the input of external energy (binding of an effector) using energy perturbation, in our recent study a new concept is developed to quantitatively describe protein dynamics in terms of energy dissipation. Furthermore, protein dynamical modules are introduced and defined based on the residue response time to bridge protein structure and function. Different from the protein structural modules which merely provide information about the structural stability of proteins, protein dynamical modules could reveal protein characteristics from the perspective of dynamics [17].

The concept of network reconstruction is widely used to understand the structure and regulation of complex biological systems like metabolic and regulatory networks [23], [24], [25], [26]. Proteins are complex residue-residue interaction (RRI) systems if we regard the amino acid residues as nodes and the residue-residue interactions as edges. Protein RRI networks have been constructed and studied in literature [27], [28], [29], [30]. However, these networks are merely constructed based on the crystal structures of proteins and not able to show the information flow within proteins. In order to explore how the signal is transferred within molecules like allosteric proteins upon binding of an effector, new concepts and methods are needed. In this work, a novel algorithm of network reconstruction is combined with the energy dissipation model to reveal the process of intramolecular signal transduction involved in allosteric regulation. A key feature of the novel approach is that directed RRI networks that involved in the process of intramolecular signal transduction could be constructed based on the data of residue response time predicted from the energy dissipation model. For this purpose, specific molecular dynamics simulations are designed and carried out to obtain residue response time during the energy dissipation process.

**Figure 1:**
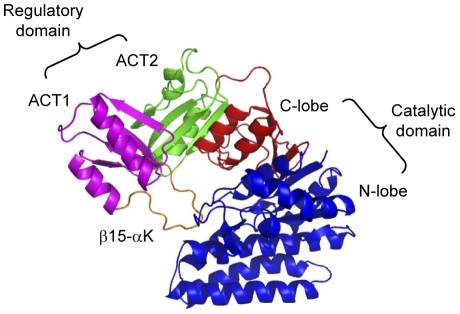
Structural regions of *E. coli* aspartokinase III. The structure is organized into a C-terminal regulatory region and an N-terminal catalytic region. The C-terminal regulatory region consists of two ACT domains, in which the second ACT domain (colored in green) is inserted within the first (colored in purple) via connections in two β-strands. ACT1 exhibits the fold of a typical ACT domain with an extended 14-residue loop between β15 and αK (colored in orange). The catalytic region exhibits a typical amino acid kinase family fold which can be further divided into the N-terminal lobe (N-lobe, colored in blue) and the C-terminal lobe (C-lobe, colored in red).

We use a well-studied allosteric protein, aspartokinase III of *Escherichia coli*, as a model system to demonstrate the new method and its usefulness. Aspartokinase is a key regulatory enzyme in the synthesis of aspartate derived amino acids. Small molecules such as lysine or threonine are separately or jointly bound at the regulatory regions, causing a change of the protein from an active state to an inactive state and the loss of enzyme activity. The regulatory domain of aspartokinase contains the ACT domain which is composed of four β strands and two α helices arranged in a βαββαβ fold (Fig. 1) and named after the first letters of three of the proteins, aspartate kinase-chorismate mutase-tyrA (prephenate dehydrogenase). This structural motif is one of a growing number of different intracellular small molecule binding domains that function in the control of metabolism, solute transport, and signal transduction [31]. *E. coli* aspartokinase III (EC 2.7.2.4) is monofunctional and allosterically inhibited by lysine [32]. The subunit is organized into a C-terminal regulatory region and an N-terminal catalytic region. The C-terminal regulatory region consists of two ACT domains, in which the second ACT domain is inserted within the first. The catalytic region exhibits a typical amino acid kinase family fold [33], which can be further divided into the N-terminal lobe (N-lobe) and the C-terminal lobe (C-lobe) (Fig. 1).

The allostery of *E. coli* aspartokinase III has been recently studied by a combined approach of statistical coupling analysis and molecular dynamics simulation (SCA-MD) [15]. Several sites have been successfully developed to alter the allostery of aspartokinase III by the effector lysine and their underlying mechanisms were investigated employing protein dynamics modules [17]. However, considering the fact that not all of the residues of these modules contribute to the allosteric communication, it is necessary to develop a novel algorithm in order to figure out those residues that contribute to the signal transduction process. The new strategy proposed in this work successfully predicted the functionally important sites that have been experimentally proved to desensitize allosteric regulation of the enzyme and revealed hereto unknown features of the allosteric communication. Moreover, our approach is not dependent on the availability of protein sequences for evolutionary analysis and provides more information than ever before. From the practical viewpoint, it will have significant impact on rational design of novel allosteric proteins once we are able to construct the signal transduction network with direction information.

### Theoretical background of the new approach

The new protein dynamics model of energy dissipation is based on the following facts:

- Protein is an open system which means energy can be transferred from external environment through intermolecular interactions (i.e. caused by a ligand binding).
- Residues in the protein are dynamic and fluctuating.
- Regulatory process is conducted by intramolecular non-linear interactions.
- The conformation of a protein with or without ligand is in a quasi-equilibrium state, whereas intermediate conformations during the signalling process are in non-equilibrium states.

According to the energy dissipation model, the allosteric process can be described as follows:

- allosteric protein is in an initial conformational distribution in which the population of the R-state (in the case of feedback inhibition) or the T-state (in the case of feedback activation) is higher than that of the others;
- external energy is introduced into the allosteric site through intermolecular interactions when the ligand binds to it;
- the input energy dissipates within the allosteric protein through intramolecular non-linear interactions;
- the allosteric protein reaches a new conformational distribution in which the population of the T-state (in the case of feedback inhibition) or the R-state (in the case of feedback activation) is higher than that of the others.

The new model represents an extension of the concept of population shift by emphasizing that after the allosteric process of the open protein system is stimulated, the energy perturbation will pass through within the protein in a dissipative pattern, resulting in a re-distribution of protein conformational states.

## Materials and Methods

**Figure 2:**
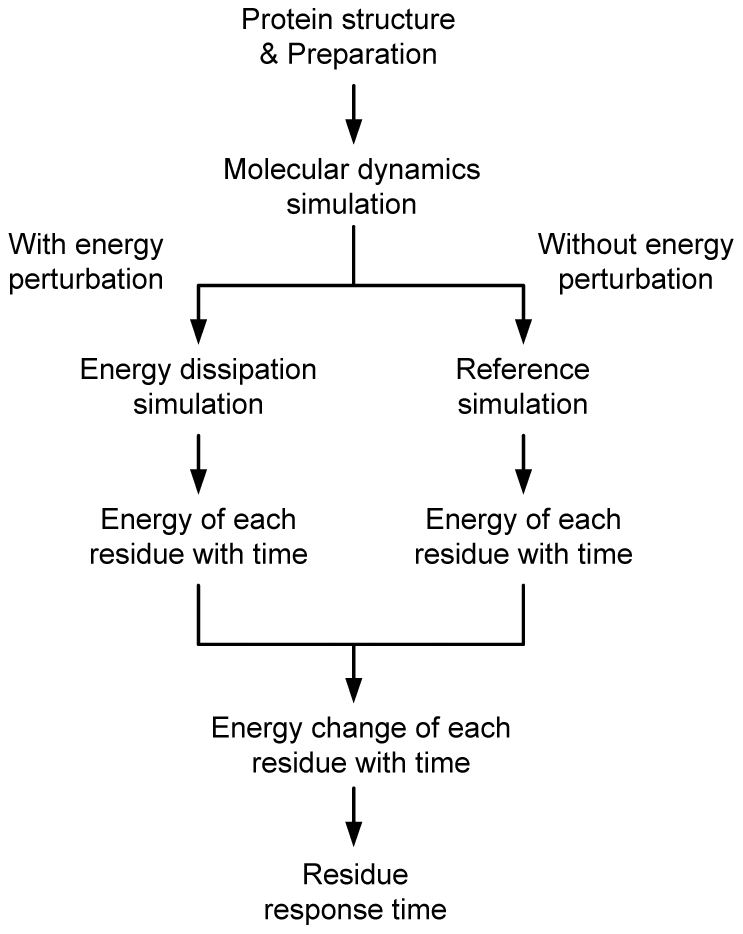
Procedures used to obtain residue response time.

Residue response time during the energy dissipation process is obtained according to the procedures given in Fig. 2 which is composed of the following steps.

### Structures

The X-ray diffraction structures of *E. coli* aspartokinase III were retrieved from Protein Data Bank (PDB) [34]. According to the model, the active state is dominant in the initial ensemble of *E. coli* aspartokinase III. Therefore, the active state could be chosen to demonstrate the dynamical process. In this work crystal structure of the R-state (PDB code: 2J0W) was employed to conduct molecular dynamics simulations followed by energy dissipation simulations as well as indentifying the key amino acid residues that interact with substrates at the catalytic site. Crystal structure of the T-state (PDB code 2J0X) was used to identify the key residues that have interactions with the effector at the regulatory site.

### Preparation

After deleting the substrates binding to the catalytic site, crystal structure of the R-state was neutralized by adding sodium and chlorine ions with an ionic concentration of 0.5 mol L^−1^ and solvated in a rectangular box of TIP3P [35] water molecules with a minimum solute-wall distance of 10 Å. The solvated systems were energy-minimized by 5,000 steps employing the software of NAMD [36] prior to the molecular dynamics simulations in order to relax the loops and side chains to make them suitable for performing the simulations.

### Molecular dynamics simulations

The aim of this step is to obtain the equilibrium conformation of the R-state protein, whose population is the highest in the initial ensemble. Molecular dynamics simulations were performed with a periodic boundary condition in the NPT ensemble using Langevin dynamics at 310 K with the damping coefficient of 5.0 ps^−1^ and constant pressure of 1 atm. The non-bond pair list was updated every 10 steps and the Particle Mesh Ewald (PME) method [37] was used to treat long-range electrostatic interactions. A residue-based cut-off of 12 Å was applied to the non-covalent interactions. No constraint was applied to the protein during the molecular dynamics simulations. A time step of 2 fs was used and the coordinates of the simulated complexes were saved every 1.0 ps. The simulations lasted 600 ps and were performed employing the software of NAMD with the CHARMM27 force field. Analysis of the molecular dynamics trajectory was conducted on the entire simulation to ensure the dynamical stability of the system. To examine the convergence of the molecular dynamics simulations, energy, temperature and pressure were monitored during simulations [17].

### Energy dissipation simulations

According to the energy dissipation model, if the energy of residues at the regulatory site is changed, the energy perturbation will be transferred to the catalytic site through intramolecular non-linear interactions. This energy dissipation process will show a unique pattern which can reflect the dynamical characteristics of the protein. However, the energy transferred to the binding pocket is conducted by forces such as van der Waals force and electrostatic force in the form of potential energy. Considering the facts that potential energy and kinetic energy transfer to each other during the process of protein dynamics and it is easy to change the kinetic energy of atoms in molecular dynamics simulations, the energy of key residues residing in the regulatory site was changed by increasing its velocity by four times in this study.

To avoid involvement of unexpected force or energy, the energy dissipation simulations were conducted with a time step of 1 fs under the condition that the temperature and pressure of the system were able to change automatically during the simulation process. Other simulation parameters were the same as that used in the former step. In the meanwhile, reference simulations in which the velocities of the residues were not changed were carried out to simulate the molecular dynamics process when no external energy was input to the protein. The energy of each amino acid residue was captured during the entire dissipation process for both simulations. Then, energy change could be calculated by subtracting the energy of the reference simulations from that of the energy dissipation simulations. With an energy change cut-off of 0.01 kcal mol^−1^, the response time of each residue caused by the energy perturbation was obtained. Both the energy dissipation simulation and the reference simulation lasted 1,000 fs when all residues had responded to the energy perturbation.

For more details about the energy dissipation model and the procedures, please refer to our recent publication [17], in which the energy dissipation model was proposed to reveal protein dynamics. Here, the focus is put on the construction of intramolecular signal transduction networks based on residue response time.

### Method for network construction and the algorithm

**Figure 3:**
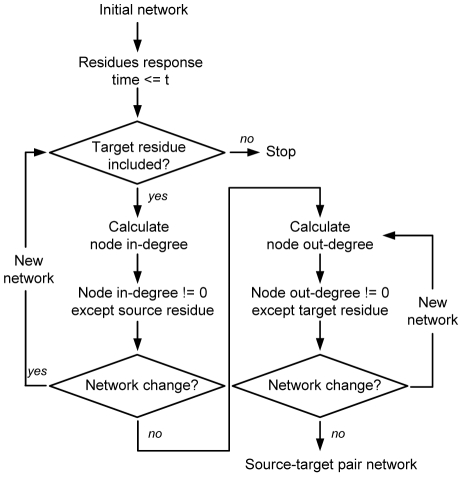
Algorithm proposed to construct source-target pair networks from the Initial network.

With a cut-off value for the distance of residue interactions, an undirected RRI network could be obtained from the crystal structure of a protein. Nodes can be represented either by the Cα or the mass center of residues. In this work, a residue is considered to interact with those whose atoms are within a cut-off distance of 6 Å. Data of residue response time obtained from the energy dissipation simulations were then put into the undirected network, resulting in a directed RRI network (referred to as the Initial network). Next, the signal transduction network from the source residue to the target residue was achieved using the algorithm which consists of three major steps (Fig. 3).

Step 1. Nodes whose response time is larger than t (the response time of the target residue) are deleted.

Step 2. Nodes whose in-degree is zero are deleted except the source residue. This step cycles until the number of nodes of the new generated network does not change any more. The value of node in-degree is re-calculated for each new network.

Step 3. Nodes whose out-degree is zero are deleted except the target residue. This step cycles until the number of nodes of the new generated network does not change any more. The value of node out-degree is re-calculated for each new network.

Usually, the regulatory site and the catalytic site are composed of several key residues that interact with ligand. Thus, the procedure was conducted for each pair of source-target residues to get the signal transduction networks from the regulatory site to the catalytic site. The obtained networks are referred to as the source-target pair networks.

### Definition of propagation coefficient

A hierarchical layout is commonly used to reveal the information flow within a directed network. For this algorithm, the nodes are placed on different layers from top to bottom, depending on directions of the edges. Once the nodes are attributed to a layer, order of the nodes is rearranged within the layers to minimize the crossings of the edges. In this work the software Cytoscape [38] was employed to obtain the hierarchical layout of the signal transduction network. From the hierarchical architecture, it can be seen that information is transferred from the higher layers to the lower layers through different pathways. To characterize the information propagation capability of a node within the signal transduction network, a new parameter of propagation coefficient is defined as follows:
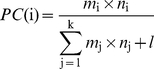
(1)where *PC*(i) is the propagation coefficient of node i that resides in layer N; k is the total number of nodes residing in layer N; *m*_i_ and *m*_j_ are the in-degree of node i and j; *n*_i_ and *n*_j_ are the out-degree of node i and j; *l* is the number of edges that pass through layer N. The denominator of the right side stands for all possible pathways from the former layer to the later layer; whereas the numerator stands for the possible pathways conduced by node i. Because the propagation coefficient takes into account not only the information of node degree but also the number of nodes residing in the layer as well as the number of edges passing through the layer, it is better than the commonly used node degree to determine the propagation capability of nodes in a directed network like the signal transduction networks. However, it is noteworthy that a deep-preference hierarchical layout should be carried out before the calculation of propagation coefficient.

## Results and Discussion

### Signal transduction network

**Figure 4:**
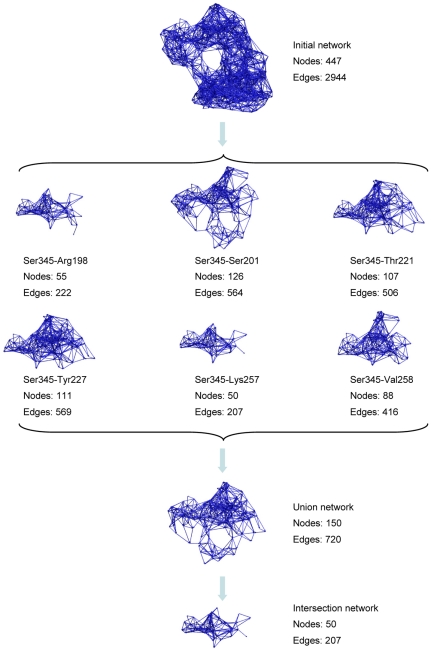
Signal transduction networks. Networks are constructed based on the new protein dynamics model of energy dissipation and the novel algorithm of network construction.

After constructing the protein RRI network with a cut-off value of 6 Å, data of residue response time obtained from the energy dissipation simulations were put into the undirected network, resulting in the Initial network (Fig. 4). The Initial network consists of all residues of *E. coli* aspartokinase III (447 nodes) and all direction information between them (2944 edges). Considering the fact that the allosteric regulation is the process of signal transduction from the regulatory site to the catalytic site, some direction information included in the Initial network may not be relevant to this process and thus could be deleted from the Initial network to achieve signal transduction network. For this purpose, residues that interact with substrates were recognized according to the crystal structure of aspartokinase III (PDB code: 2J0W). With these residues as targets, signal transduction networks for each source-target pair were obtained from the Initial network employing the algorithm of network construction described in Materials and Methods.

As shown in Fig. 4, the source-target pair networks consist of different number of nodes and edges. The smallest network (residue pair of Ser345-Lys257) has only 50 nodes and 207 edges, whereas the largest one (residue pair of Ser345-Ser201) has 126 nodes and 564 edges. This implies that some pathways may be common among them, whereas some may be unique. Table 1 lists the number of common residues among the source-target pair networks. For the largest network, 37 residues are unique; while the others are shared with the other pair networks. The smallest network shares all its residues with the other pair networks.

**Table 1:** Number of residues that influence different number of target residues for each source-target pair network.

	Number of target residues					
Source-target pair networks	1	2	3	4	5	6
Ser345-Arg198	0	1	0	0	4	50
Ser345-Ser201	37	1	0	34	4	50
Ser345-Thr221	1	18	0	34	4	50
Ser345-Tyr227	5	18	0	34	4	50
Ser345-Lys257	0	0	0	0	0	50
Ser345-Val258	0	0	0	34	4	50

**Figure 5:**
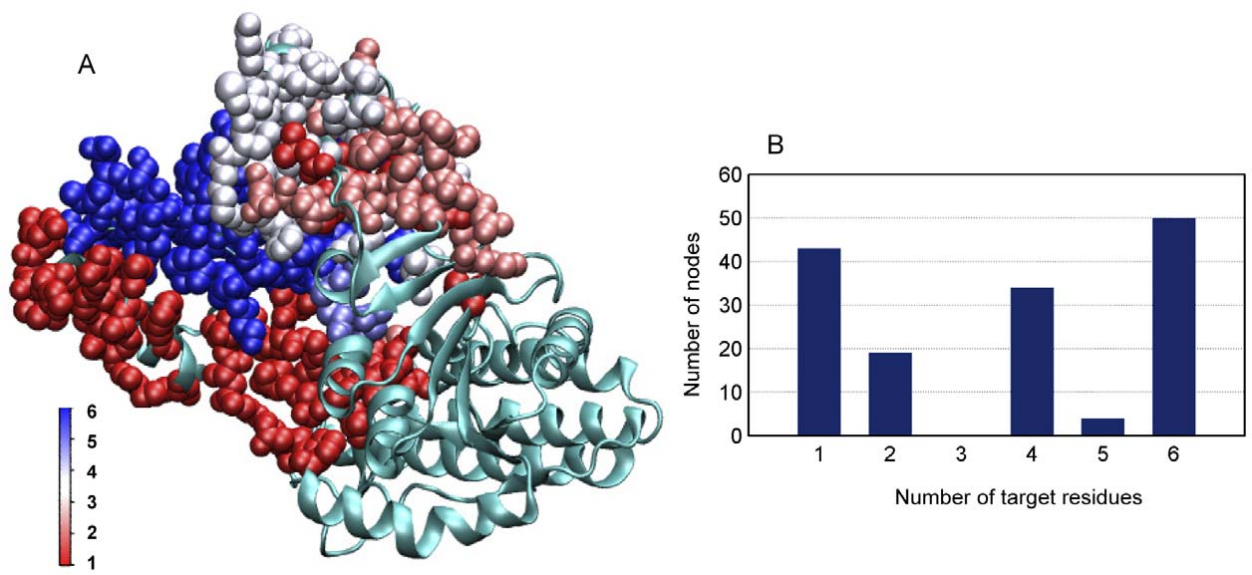
The Union network. (A) Residues are colored according to the number of target residues they can influence. (B) The number of nodes that can affect different number of target residues.

Merging all of the source-target pair networks together results in the “Union network” (Fig. 4 and 5A). The Union network is composed of all the possible signaling pathways from the regulatory site to the catalytic site. Change of any residue in the Union network would influence the process of signal transduction. However, the underlying mechanism may be different. Some changes may influence the signal transduction to only one of target residues without the influence on the others; some changes may affect two or more target residues at the same time (Fig. 5B). Obviously, residues that are involved in all of the pair networks (blue-colored in Fig. 5A) are more significant than the others. Getting together those residues we obtained the “Intersection network” (Fig. 4). Considering the importance of the residues composing the Intersection network, the Intersection network can be regarded as the core of the Union network. However, it is not proper to regard the Intersection network as the smallest network that ensures the process of signal transduction, because nodes not in the Intersection network but in the Union network, can also influence the signaling process, which can be seen from the experimental results of such nodes as shown in the following sections.

Table 2 lists the basic network parameters for the Initial network, the Union network and the Intersection network. Associated with the decrease of the numbers of nodes and edges within each network, the network diameter, the characteristic path length and the average number of neighbors also decrease. In contrast, the percentage of shortest path and the clustering coefficient increase and the network radius keeps constant for all of them. It is clear that a core signal transduction network which is around ten percentage of the Initial network was successfully inferred using the algorithm of network construction.

**Table 2:** Network parameters of the signal transduction networks (calculated using Cytoscape [38]).

Parameters	Initial network	Union network	Intersection network
Number of nodes	447	150	50
Number of edges	2944	720	207
Network radius	1	1	1
Network diameter	15	10	7
Characteristic path length	4.724	3.534	2.544
Shortest paths	38329 (19%)*	6063 (27%)	916 (37%)
Average number of neighbors	13.172	9.600	8.280
Clustering coefficient	0.247	0.255	0.284

*: Percentage of the shortest paths in all possible paths.

### Comparisons of residue distribution

**Figure 6:**
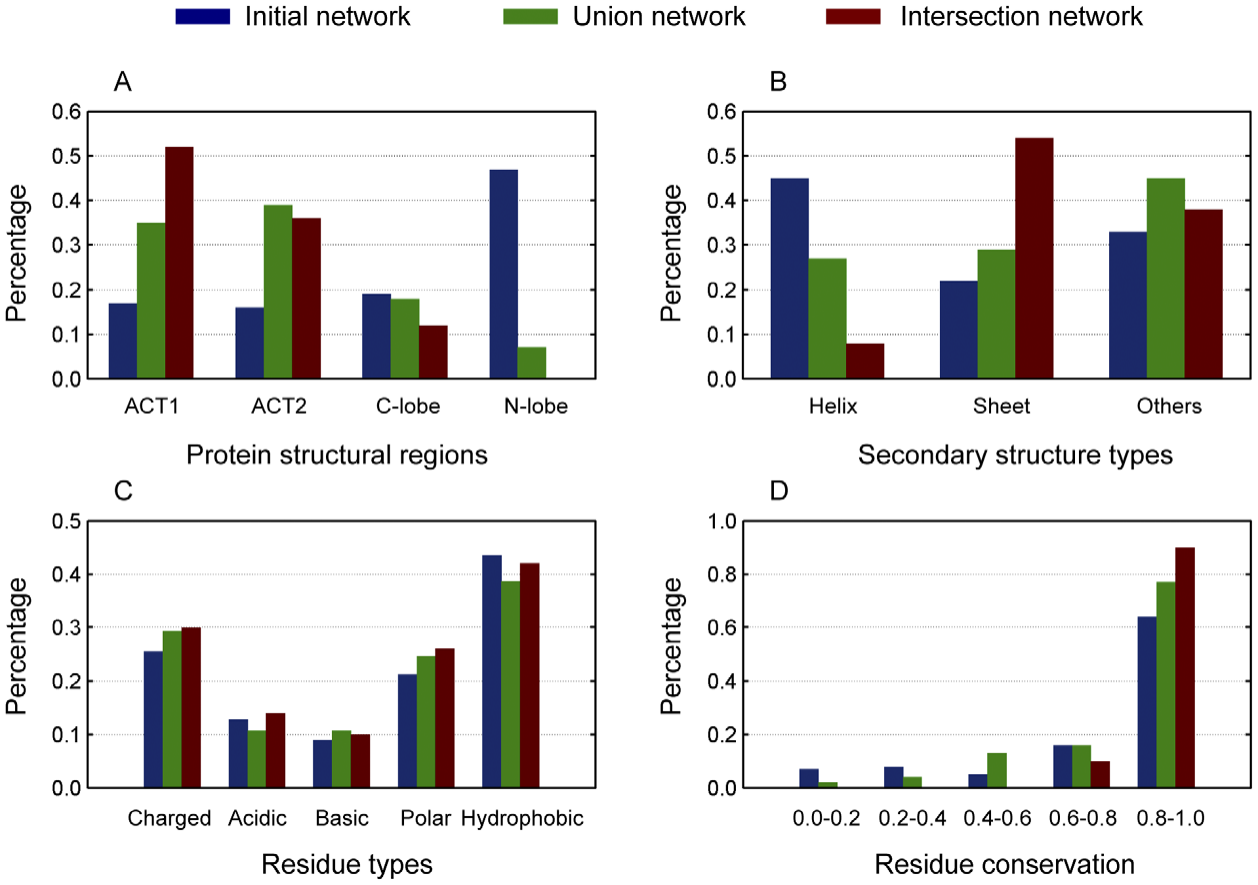
Comparisons of residue distribution for the Initial network, the Union network and the Intersection network. (A) protein structural regions, (B) secondary structure types, (C) residue types, (D) residue conservation.

In order to better understand the features utilized by the *E. coli* aspartokinase III when constructing its signal transduction network, several comparisons of residue distribution were carried out for the Initial network, the Union network and the Intersection network. In the case of residue distribution among protein structural regions (Fig. 6A), more residues reside in the regulatory domain (ACT1 and ACT2) than that in the catalytic domain (C-lobe and N-lobe) with the decrease of network size. Nearly half of the residues of the Initial network reside in the N-lobe region, whereas almost half of the residues of the Intersection network belong to the ACT1 region with none in the N-lobe region. This distribution characteristic indicates that the protein dynamical property involved in the process of signal transduction is mainly conducted by the regulatory domain and that the role of the catalytic domain is merely to realize its catalytic function. Thus, in practice it is possible to design lysine-sensitive allosteric proteins by introducing the regulatory domain of *E. coli* aspartokinase III to a non-allosteric protein.

As regard to the distribution of secondary structure types (Fig. 6B), nearly half of the residues of the Initial network are the Helix type, whereas over half of the residues of the Intersection network are the Sheet type. Considering the residue distribution among protein structural regions and the fact that the regulatory domain is mainly composed of the secondary structure of Sheet type whereas the catalytic domain mainly consists of Helix type, it is not difficult to understand the distribution result among the secondary structure types. It is noteworthy that, for all of the networks, secondary structure types of the “Others” group, such as loops, also play an important role in the signal transduction process. In Fig. 6C, amino acid residues are divided into five different groups according to their polarization. It is a little surprising that no evident preference could be observed for the signal transduction networks. This means that polar and non-polar residues contribute equally to the signal transduction process. At least this is true for the case of *E. coli* aspartokinase III.

Another important distribution is about the residue conservation (Fig. 6D). It is well known that residues which play significant roles in protein structures and functions are better conserved during the evolution of proteins. This feature has been widely used to construct communication network for allosteric proteins to figure out key residues [14], [39]. Since the networks constructed based on the new model and algorithm is able to reflect the signaling process, residues of the signal transduction networks should also show high conservation. As it is expected, Fig. 6D clearly shows that ninety percent of the residues of the Intersection network have the conservation of more than 0.9 with the others between 0.6 and 0.8. This result illustrates the high conservation of the core signal transduction network during protein evolution and demonstrates the powerful ability of the new approach to figure out conservation residues.

### Super-hubs and motif preference

**Figure 7:**
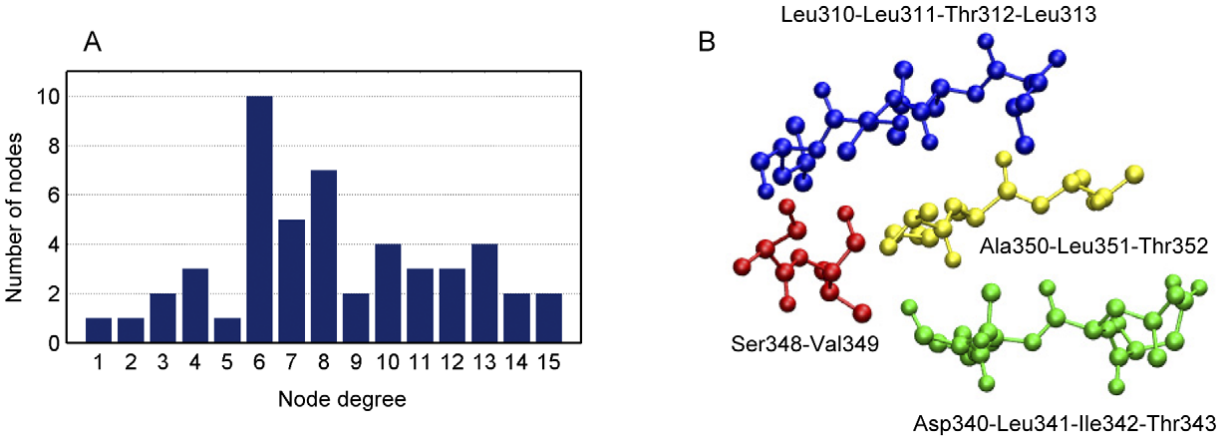
The core signal transduction network. (A) Node degree distribution. (B) Three-dimensional positions of the super-hubs.

The node degree distribution was calculated for the core signal transduction network of *E. coli* aspartokinase III (Fig. 7A). Nearly half of the nodes have 6–8 neighbors. When we focus on the nodes with high node degrees (>10), it is interesting to find that these residues tend to form super-hubs: Leu310-Leu311-Thr312-Leu313, Asp340-Leu341-Ile342-Thr343, Ala350-Leu351- Thr352 and Ser348-Val349. Three-dimensional structures of these super-hubs are given in Fig. 7B. Discovery of super-hubs in signal transduction network indicates that allosteric protein tends to gather residues with high connection ability to facilitate the signaling process and to increase the robustness of the signal transduction network against external influences.

**Figure 8:**
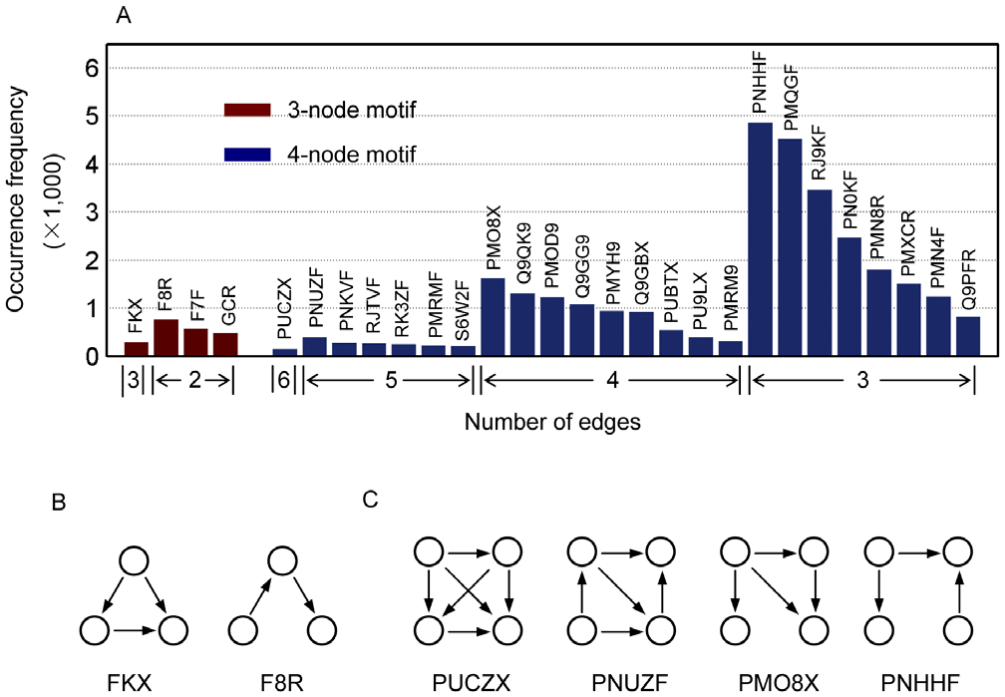
Motif occurrence frequency of the core signal transduction network. (A) Motifs are arranged first by their edges then by their occurrence frequency. The 3-node (B) and 4-node (C) motifs with the highest occurrence frequency for different edges. Motif occurrence frequency was calculated using MAVisto [40].

Different from hubs, motifs are linkage patterns utilized by networks to organize its structure. Networks with different physical meanings will normally exhibit different motif preference. Thus, it is a useful parameter in network analysis to reveal the construction characteristic of a network, especially the 3-node and the 4-node motifs. Here, motif search was carried out for the core signal transduction network of *E. coli* aspartokinase III (Fig. 8A). In the search of 3-node motifs, motif FKX and F8R show the highest occurrence frequency for three and two edges separately (Fig. 8A and B). Similarly, motif PUCZX, PNUZF, PMO8X and PNHHF show the highest occurrence frequency in the case of the 4-node motifs (Fig. 8A and C). Occurrences of motif FKX in the 3-node motif and motif PUCZX, PNUZF, PMO8X in the 4-node motif illustrate the diversity and complexity of signal transduction pathways during the process of allosteric communication and stimulate the proposal of a new parameter to determine the significance of a residue in the signal transduction network.

### Characterization of propagation capability

**Figure 9:**
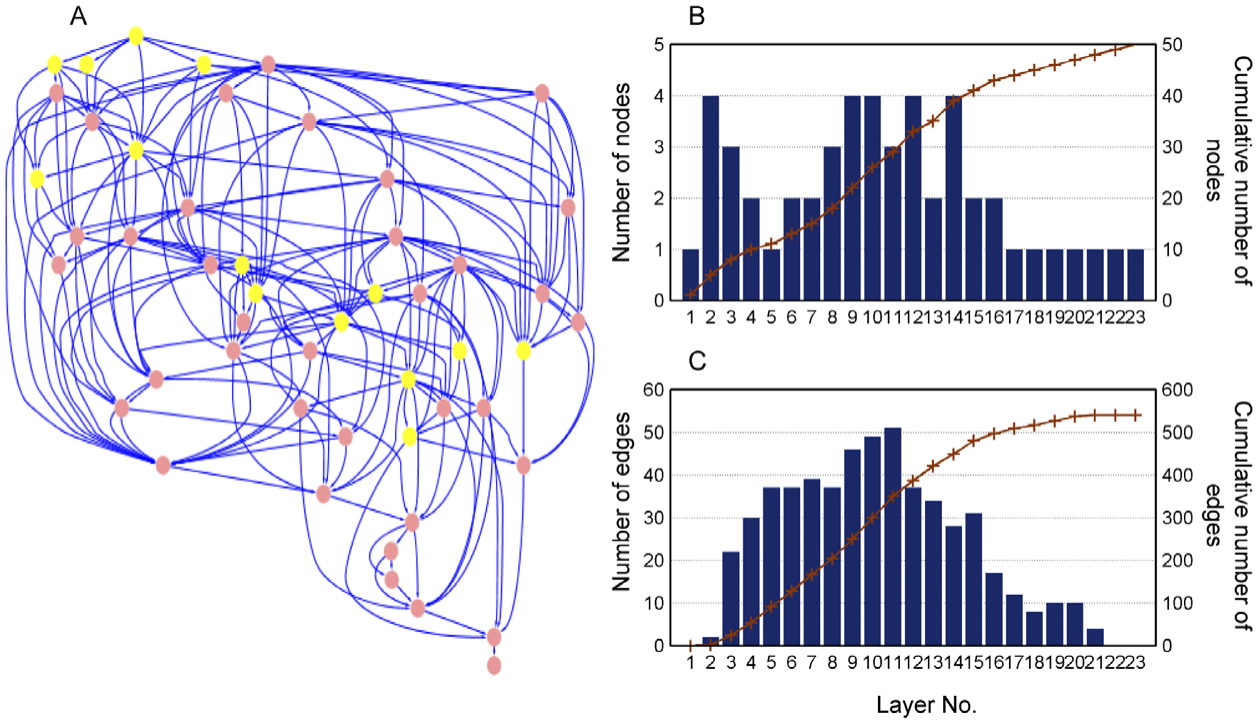
Hierarchical layout of the core signal transduction network. (A) Residues that have been experimentally proved are colored in yellow. (B) The number of nodes for each layer (histogram) and the cumulative number of nodes (line). (C) The number of edges passing through each layer (histogram) and the cumulative number of edges (line).

As an important feature of directed network, it is possible to examine its hierarchical architecture. When the signal transduction network is hierarchically layout with deep-preference using the software Cytoscape [38], it is clear to see the information flow that is carried by the energy change of the residues (Fig. 9A). It can be seen that the hierarchical architecture is composed of many layers. Each layer consists of several nodes and edges that pass though it. The total number of layers represents the longest pathway from the regulatory site to the catalytic site. Nodes receive signals from a higher layer and send them to a lower layer. Thus, each layer contains all the possible pathways for signals to pass through from the higher layer to the lower layer.

The information flux passing through a node reflects its propagation capability. Characterizing the propagation capability of a node is not only meaningful to interpret the signal transduction process but also useful for rational modification of allosteric proteins. Although node degree is able to measure the connection ability of a node, it is not suitable for determining the propagation capability of a node in a directed network like the signal transduction network. That is because the number of nodes residing in the layer and the number of edges that pass through the layer should also be taken into account to calculate the possible signaling pathways conducted by a certain layer. The large number of nodes in each layer (Fig. 9B) and edges passing through each layer (Fig. 9C) indicates that many other pathways could propagate signals and thus maintain the protein function when one or some of them are disturbed (e.g. by mutagenesis). Thus, it is necessary to take them into account to measure the propagation capability of a node.

**Figure 10:**
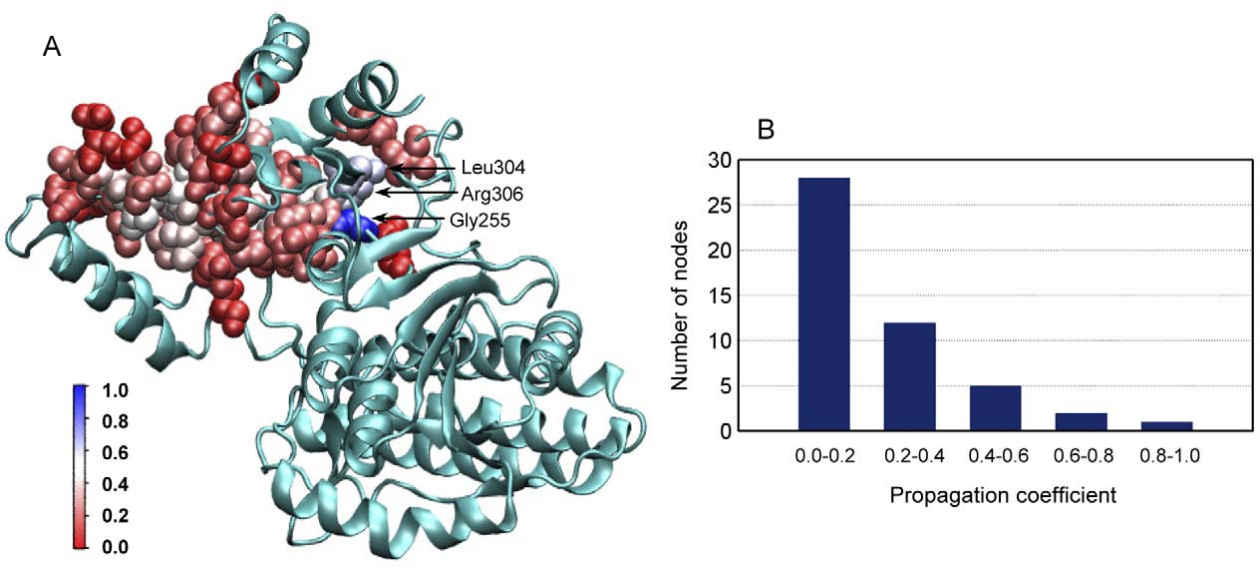
Residue propagation coefficient and its distribution for the core signal transduction network. (A) Residues are colored according to its propagation coefficient. (B) The propagation coefficient distribution of the nodes.

A new parameter, propagation coefficient (*PC*) (Eq. 1), is proposed in this work. *PC* is defined as the percentage of pathways that pass through a node of a certain layer. Therefore, a larger value of *PC* indicates that more signals are propagated by the corresponding node and thus this node plays a more important role than the others in the signaling process. It is noteworthy that although *PC* is given in the context of layers rather than the whole network, *PC*s of nodes from different layers can be compared with each other. In Fig. 10A, the residues belonging to the core signal transduction network are colored according to their *PC*s and the distribution is shown in Fig. 10B. It can be seen that over half of the residues have a propagation coefficient smaller than 0.2. The residue with the largest propagation coefficient is Gly255 flowed by Arg306 and Leu304 (Fig. 10A).

### Prediction of functionally important sites

Functionally important sites are those residues by disturbance of which (e.g. by amino acid mutagenesis) the allosteric properties can be altered to reduce the strength of feedback inhibition by the product. Thus, any residues that play a role in the signal transduction process are functionally important sites and thus could be potential mutation sites. According to the Union network, as many as 150 residues could be the potential mutation sites. However, their roles in the signaling process may be different. Nodes with more target residues are more important than the others. Therefore, residues belonging to the Intersection network are better candidates than the others. Residues of the Intersection network can be further arranged according to their different *PC*s.

Here, the functionally important sites predicted by the new approach are compared with those that have been experimentally verified (Table 3). It can be seen that the Union network, which combines all possible pathways together and thus presents the signal transduction network from the regulatory site to the catalytic site, includes all the experimentally proved sites. To test the statistical significance of this result, the occurrence probability of the result was calculated using the following formula:
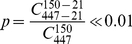
(2)where *p* is the occurrence probability; 
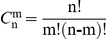
 is the combination operator; 447 is the number of residues in the Initial network; 150 is the number of residues in the Union network; 21 is the number of mutation sites that have been experimentally proved. As an alternative approach to test the statistical significance, the hypergeometric distribution method was used to calculate the occurrence probability:
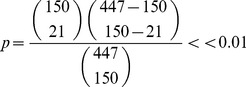
(3)where *p* is the occurrence probability; 
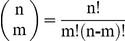
 is the binomial coefficient; meanings of the numbers are the same as above. Two methods got the same result. The small value of the occurrence probability indicates that the result shown here is not a chance.

**Table 3:** Comparison of mutation sites predicted by the new approach with those reported in literature.

Mutation sites	Number of target residues*	*PC*	Reference	Mutation sites	Number of target residues	*PC*	Reference
M251P	4	-	[15]	E346R	6	0.04	[15]
T253R	6	0.17	[15]	V347M	6	0.19	[41]
R305A	6	0.42	[15]	V349M	6	0.53	[41]
S315A	6	0.11	[15]	T352I	6	0.50	[15]
M318I	1	-	[41]	T355	1	-	-
H320A	1	-	[15]	C378	1	-	-
G323D	1	-	[41], [42]	I392	1	-	-
F324	1	-	-**	F407	4	-	-
L325F	1	-	[41]	N414	0	-	-
F329R	1	-	[15]	R416A	6	0.10	[15]
I337P	1	-	[15]	M417I	6	0.20	[41]
S338L	6	0.03	[15]	S423	6	0.09	-
V339A	6	0.10	[15]	S424	4	-	-
T344M	6	0.19	[42]	N426	4	-	-
S345L	6	1.00	[41], [42]	C428R	4	-	[15]

*: Number of target residues the corresponding mutation site can influence.

**: Mutation sites that have not been experimentally proved.

### Comparison with the SCA-MD method

The statistical coupling analysis (SCA) is a powerful approach to define the architecture of functional interactions between amino acids and can help understanding the basic physical principle underlying protein structure, function, and evolution by extending the traditional definition of conservation to include correlations between positions [39]. Application of this method to structurally and functionally distinct protein families reveals a surprisingly simple architecture for amino acid interactions in each protein family [43]. Molecular dynamics simulations and SCA data were combined (SCA-MD method) to identify residues that are important for catalysis [44]. In a recent study from our group, the SCA-MD method was employed to guide engineering allosteric regulation of *E. coli* aspartokinase III [15]. The allosteric properties were altered as desired to reduce the strength of feedback inhibition by the product(s). However, the co-evolutionary approach depends on the quantity and quality of sequence information obtained for the protein family. Moreover, it does not provide information for the underlying mechanisms of regulation.

When the functionally important sites predicted by the new approach are compared with those given by the SCA-MD method, it is found that the Union network contains all of the 30 sites given by Chen et al. [15] except N414 (Table 3). In the meanwhile, it is not surprising to see that these sites influence different number of target residues. Only half of the 30 sites, which have 6 target residues, belong to the core signal transduction network (Fig. 8A, yellow circles). Their *PC*s range from 0.03 for S338L to 1.0 for S345L. The high agreement of the results from the new strategy with that based on the co-evolution of sequence information is not difficult to understand if we look again on the comparison of their residue conservation distributions (Fig. 5D). Nevertheless, an advantage of the new approach proposed in this work is that it is not only able to reveal residues with high conservations but also able to distinguish residues that are conserved for the allosteric communication from those which are kept to realize the catalytic function (Fig. 5A).

### Conformational state-sensitivity of the signal transduction networks

Allosteric proteins undergo structural fluctuations over a wide range of timescales. Structural fluctuations over the wide range of timescales can be represented as different conformational states in an ensemble. The energy dissipation model employs the active states to demonstrate the dynamical process due to the fact that the population of the active states is higher than that of the others in the initial ensemble. To illustrate the conformational state-sensitivity of the intramolecular signal transduction networks, an extreme example, which used the T-state of the allosteric protein as the initial structure, is presented here using the same simulation procedures and network construction approach. Comparing the networks (Table 2 and 4), it can be seen that small differences exist although the network radius and diameter are the same as those based on the R-state of the protein. For example, the number of edges is slightly larger for the Initial network, indicating that the conformation of the T-state is more constrictive than that of the R-state. The number of nodes and edges are smaller for the Union network, whereas they are slightly larger for the Intersection network. This means although less residues contribute to the process of intramolecular signal transduction, more residues involve in all of the source-target pair networks. The conformational state-sensitivity of the signal transduction networks indicate that on the one hand, useful information can be obtained from these differences (such as in the comparison of conformation change); on the other hand, it is better to generate the signal transduction networks based on the same conformational states (for instance, the active states) when different allosteric proteins are compared.

**Table 4:** Network parameters of the signal transduction networks using the T-state as the initial structure (calculated using Cytoscape [38]).

Parameters	Initial network	Union network	Intersection network
Number of nodes	447	123	54
Number of edges	2954	627	224
Network radius	1	1	1
Network diameter	15	10	7
Characteristic path length	4.151	3.383	2.473
Shortest paths	29979 (15%)*	5115 (34%)	984 (34%)
Average number of neighbors	13.217	10.195	8.296
Clustering coefficient	0.251	0.264	0.268

*: Percentage of the shortest paths in all possible paths.

### Conclusions

In this work we used residue response time obtained from a new dynamics model of energy dissipation to provide direction information of interactions and thus to solve a major problem in constructing RRI networks of proteins. Signal transduction networks were successfully predicted from the Initial network employing a novel algorithm of network construction. In addition, a new parameter of propagation coefficient was proposed to reflect the propagation capability of a residue during the signaling process. Validation of the new method was demonstrated using *E. coli* aspartokinase III as a model system. Besides the powerful ability in prediction of functionally important sites, characteristics and mechanisms involved in the process of signal transduction were also revealed. The new approach may be applicable to other signaling molecules that are important in biotechnology and biomedicine.
